# Chemical Functionalization of Cellulose Nanofibrils with 2-Aminoethyl Hydrogen Sulfate

**DOI:** 10.1021/acsomega.4c08573

**Published:** 2024-12-23

**Authors:** Marcus Felippe de Jesus Barros, Samir Leite Mathias, Henrique Solowej Medeiros Lopes, Marcelo de Assumpção Pereira da Silva, Robson Valentim Pereira, Aparecido Junior de Menezes

**Affiliations:** † Graduate Program in Materials Science, Federal University of São Carlos—UFSCar, 18052-780 Sorocaba, São Paulo, Brazil; ‡ Multidisciplinary Institute of Chemistry, Federal University of Rio de Janeiro—UFRJ, 27930-560 Macaé, Rio de Janeiro, Brazil; § Technological College of Sorocaba—Fatec, 18013-280 Sorocaba, São Paulo, Brazil; ∥ Institute of Physics of São Carlos, University of São Paulo—USP, 13566-590 São Carlos, São Paulo, Brazil; ⊥ Central Paulista University Center—UNICEP, 13563-470 São Carlos, São Paulo, Brazil

## Abstract

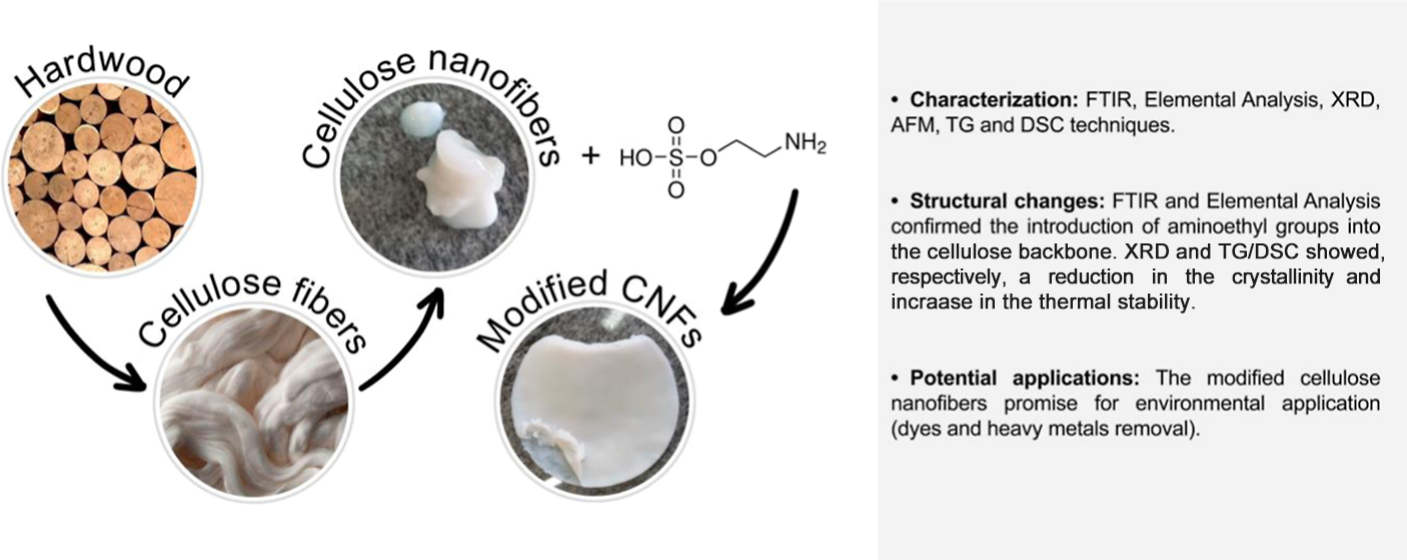

The chemical functionalization of cellulose nanofibrils (CNFs) was carried out using 2-aminoethyl hydrogen sulfate as the reagent under various experimental conditions via a bimolecular nucleophilic substitution (S_N_2) reaction. The functionalized CNFs were characterized by Fourier transform infrared spectroscopy-attenuated total reflectance. The results indicate that the chemical modification was successful, as evidenced by the presence of a band at 1540 cm^–1^, corresponding to the N–H bond of the amine group. Elemental analysis revealed a nitrogen content of 0.45%, and the degree of substitution was calculated to be 0.053 under the optimal reaction conditions. Atomic force microscopy analysis showed no significant changes in the morphology of the CNFs. X-ray diffraction patterns demonstrated a decrease in the crystallinity index, from 80.8% to 71.8%. Thermogravimetric analysis showed a slight reduction in thermal stability (onset temperature decreased from 229.4 to 227.5 °C) for the modified CNFs compared to the unmodified samples. Differential scanning calorimetry results indicated no significant effect of the modification on thermal behavior, with both modified and unmodified samples displaying similar thermal profiles, although the modified samples exhibited slightly higher thermal stability.

## Introduction

The quest to develop environmentally friendly functional materials has sparked significant interest in cellulose due to its biodegradability, biocompatibility, and nontoxic nature. Cellulose possesses a wide range of advantageous properties, including mechanical strength, thermal stability, rheological versatility, optical, low density, high aspect ratio, and a substantial specific surface area, making it a versatile material for various applications.^1−12^ Among natural polymers, cellulose is the most abundant on earth’s crust, forming a key component of the cellular structures of plants, fungi, algae, tunicates, and even certain bacterial species. Its primary function in these systems is to provide structural support and protection. Structurally, cellulose is composed of repeating units of β-1,4-glycosidically linked glucose molecules, forming a linear polymeric chain rich in hydroxyl functional groups and exhibiting a semicrystalline nature.^1−15^

Cellulose can be reduced to the nanoscale through chemical, enzymatic, and mechanical treatments, obtaining nanocellulose. Nanocellulose exists primarily in two forms: cellulose nanofibrils (CNFs) and cellulose nanocrystals. The extraction of nanocellulose from plant fibers follows a top-down deconstruction approach, which involves purification steps, such as alkaline treatment and bleaching. The bacterial cellulose is synthesized via a bottom-up approach through microbial processes.^1−17^

Owing to its intrinsic properties, nanocellulose finds applications in diverse fields, including paper production, textiles, packaging, bioplastics, composite materials, adhesives, paints, coatings, food additives, emulsions, foams, hydrogels, cosmetics, biomedicine, sensors, optoelectronics, and filtration/separation membranes.^1−19^

The high density of hydroxyl functional groups, particularly primary alcohol groups, makes cellulose highly reactive, allowing for a variety of chemical modifications. Such modifications can enhance existing material properties or introduce new functionalities. Examples of chemical modification reactions include acetylation, alkalization, amidation, esterification, etherification, and oxidation, as well as plasticization, phosphatation, silylation, and surfactant incorporation.^4−7,10,12,14,15^

This study focuses on the etherification of CNFs using 2-aminoethyl hydrogen sulfate (2AHS) as the modifying agent. The aim is to contribute to the body of research on nanocellulose chemical modification and explore its potential for developing filtration membranes capable of removing dyes and heavy metals. Notably, 2AHS was employed by Jakubovic (1959)^20^ to introduce amine groups into cellulose fibers and, to the best of our knowledge, has not yet been used in nanocellulose functionalization.

## Materials and Methods

### Materials

The 3% aqueous suspension of CNFs used in this study was supplied by Suzano S.A. Sodium hydroxide (NaOH) and 2AHS (98%) were obtained from Sigma-Aldrich and used without further purification.

### Chemical Functionalization

To functionalize the CNFs, the hydroxyl groups were first activated by treatment with a 2.6% sodium hydroxide solution at room temperature for one h under magnetic stirring with a reflux condenser in place.^21−24^ Subsequently, the etherification reaction was conducted by adding the modifying agent 2AHS dropwise to the activated CNFs at a molar ratio of [2AHS]:[OH_cel_] = 1. The reaction was carried out under reflux with stirring in an inert nitrogen atmosphere.

**Figure 1:**
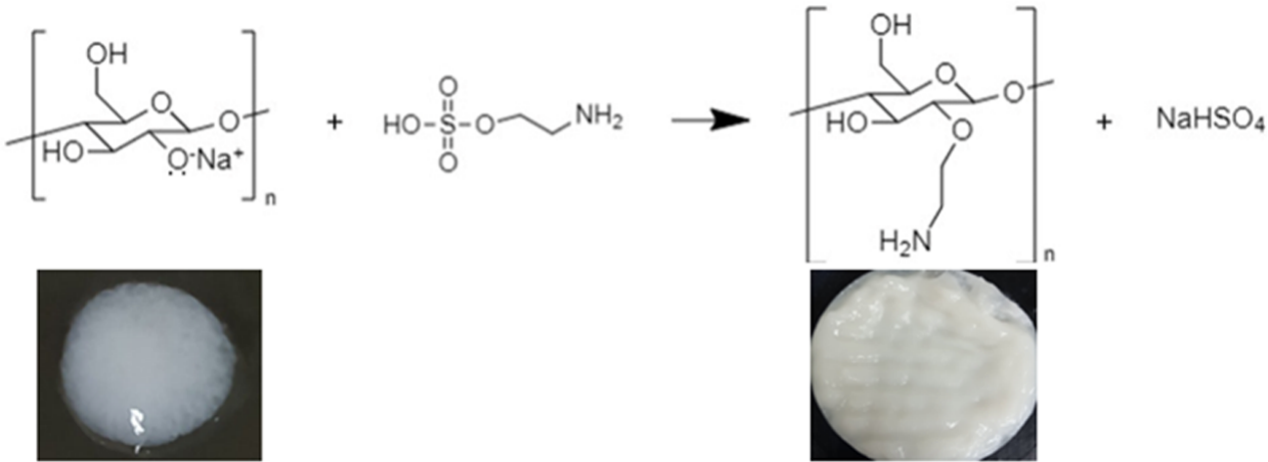
Reaction scheme for the etherification of CNFs using the 2AHS modifier.

After the chemical modification, the resulting samples were thoroughly washed with deionized water to remove electrolytes and oven-dried at 70 °C for 24 h. Figure 1 illustrates the CNF modification reaction.

### Chemical Characterization

An experimental design of 2^2^ was implemented, resulting in four experiments where the reaction factors—time and temperature—were varied. The experimental conditions for the samples are listed in Table 1.

**Table 1:** Experimental Conditions for the Reaction

samples	etherifying reagent	time (h)	temperature (°C)
CNFs			
1N	2AHS	1	50
3N	2AHS	2	50
5N	2AHS	1	100
7N	2AHS	2	100

#### Fourier Transform Infrared Spectroscopy

To evaluate the success of the reaction, Fourier transform infrared spectroscopy-attenuated total reflectance (FTIR-ATR) measurements were performed on all samples. Dried samples were analyzed by using a PerkinElmer Spectrum 65 spectrometer. The spectra were obtained at a resolution of 4 cm^–1^ with 64 scans across the 4000–500 cm^–1^ range.

#### Elemental Analysis

Performed in triplicate using a FlashSmart elemental analyzer (Thermo Scientific), we quantified the carbon, nitrogen, hydrogen, and oxygen contents of the samples.

The nitrogen content obtained from elemental analysis (EA) was used to calculate the reaction yield by estimating the degree of substitution (DS) using eq 1.^25^ The DS quantifies the proportion of hydroxyl groups substituted per glucose unit in the polymer chain, indicating the extent of amine group incorporation.^26^
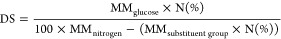
1where MM_glucose_ is the molar mass of glucose monomer (162 g·mol^–1^), N (%) is the nitrogen content determined by EA, MM_nitrogen_ is the molar mass of the nitrogen atom (14 g·mol^–1^), and MM_substituent group_ is the mass molar of the substituent group −CH_2_–CH_2_–NH_2_ (44 g·mol^–1^).

#### Atomic Force Microscopy

Micrographs were obtained using a Bruker Dimension Icon microscope with ScanAsyst mode. Aqueous solutions of the samples (0.05%) were prepared, and two drops were applied to mica substrates, which were then dried at room temperature for 24 h. The micrographs were analyzed with NanoScope Analysis software, and nanofibril diameters were measured (50 measurements) using height profiles to avoid the effect of the atomic force microscopy (AFM) tip.

#### X-ray Diffraction

X-ray diffraction (XRD) was performed using a Shimadzu LabX XRD-6100 diffractometer. Samples were previously dried in a desiccator, and measurements were conducted under the following conditions: 40 kV, 30 mA, and a scanning speed of 4°/min over a 2θ range of 10–30° using Cu Kα radiation (λ = 0.15406 nm).

The crystallinity index (CI) was calculated using Segal’s method (eq 2).^27^
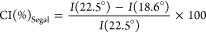
2where *I*(22.5°) corresponds to the intensity of both crystalline and amorphous regions, while *I*(18.6°) corresponds to the intensity amorphous region.^27^

#### Thermogravimetry

Thermal behavior was assessed using a PerkinElmer Pyris thermogravimetric analyzer. Samples (5–6 mg) were analyzed in aluminum pans at a heating rate of 10 °C·min^–1^ from 25 to 600 °C under a N_2_ flow of 20 mL·min^–1^.

#### Differential Scanning Calorimetry

Thermal transitions were evaluated using a TA Instruments DSC 25 equipped with a Refrigerated Cooling System (RCS 90). Samples (5–6 mg) were sealed in hermetic pans and preheated to 120 °C for 5 min to remove residual moisture and thermal history. The temperature range for the second heating scan was −90 to 250 °C, with a heating rate of 10 °C·min^–1^ under a nitrogen flow of 50 mL min^–1^.

## Results and Discussion

### Fourier Transform Infrared Spectroscopy

**Figure 2:**
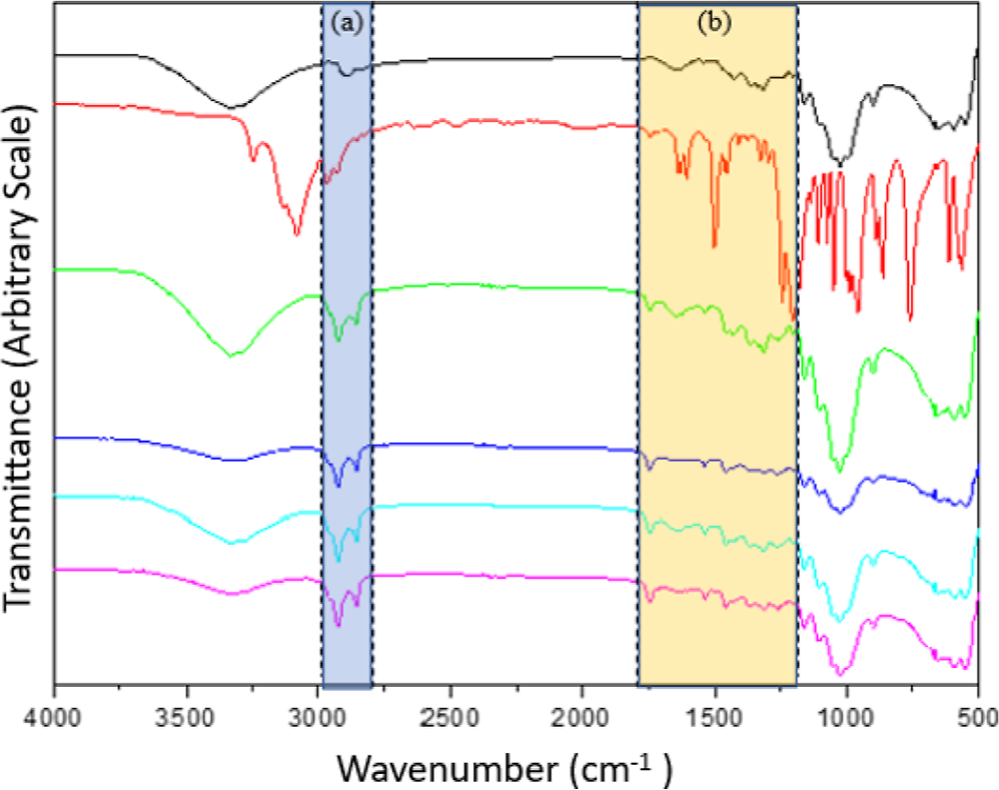
FTIR spectra of the samples: (black) CNFs; (red) 2AHS; (green) 1N; (blue) 3N; (sky blue) 5N; (violet) 7N.

Figure 2 illustrates the characteristic bands of cellulose in the FTIR-ATR spectrum. The stretching around 3500 cm^–1^ and 3200 cm^–1^ is attributed to hydroxyl (OH) groups. Peaks near 2800 cm^–1^ and 1430 cm^–1^ correspond to asymmetric and scissor stretching of the methylene bonds, respectively. The band at 1320 cm^–1^ is associated with the flexion of OH groups, while those between 1200 cm^–1^ and 1400 cm^–1^ represent CH_2_ stretching within the pyranose ring. The region between 900 cm^–1^ and 1200 cm^–1^ exhibits high absorption and overlapping signals related to C_6_–O and pyranose C–O–C stretching, while the peak at 890 cm^–1^ corresponds to glycosidic bond stretching.^28−30^

The key vibrational bands of the 2AHS reagent are also highlighted in Figure 2. A broad region between 3250 and 3131 cm^–1^ corresponds to the aliphatic amino group. Peaks at 2967 cm^–1^ and 2927 cm^–1^ are associated with CH_2_ bonds. A medium-to-strong-intensity band between 1635 and 1609 cm^–1^ is attributed to symmetric angular deformation of NH_2_. Bands in the range of 1466–1455 cm^–1^ correspond to angular deformation of CH_2_. The region between 1300 and 1250 cm^–1^ is indicative of C–N stretching. Peaks in the 910–660 cm^–1^ range are associated with out-of-plane angular deformation of amine groups and near 757 cm^–1^ represent asymmetric angular deformation of CH_2_.^29,31^

To identify aliphatic primary amines, the main absorption bands in the infrared spectrum include (i) two medium-intensity bands near 3555 cm^–1^ and 3300 cm^–1^, corresponding to asymmetric and symmetric N–H stretching, respectively, which may shift to higher wavenumbers due to hydrogen bonds; (ii) a band near 2780 cm^–1^ attributed to N–CH_2_ stretching; (iii) bands in the range of 1640–1550 cm^–1^, representing in-plane angular deformation of H–N–H; (iv) a band between 1300 and 1250 cm^–1^, assigned to C–N stretching, and this band is difficult to visualize due to the presence of aliphatic ethers in cellulose, but changes in its intensity are indicative of successful grafting; (v) the region 1260–1020 cm^–1^, associated with C–N stretching vibrations; and (vi) a low-intensity band between 850 and 600 cm^–1^, attributed to out-of-plane angular deformation of NH_2_.^29,31^

**Figure 3:**
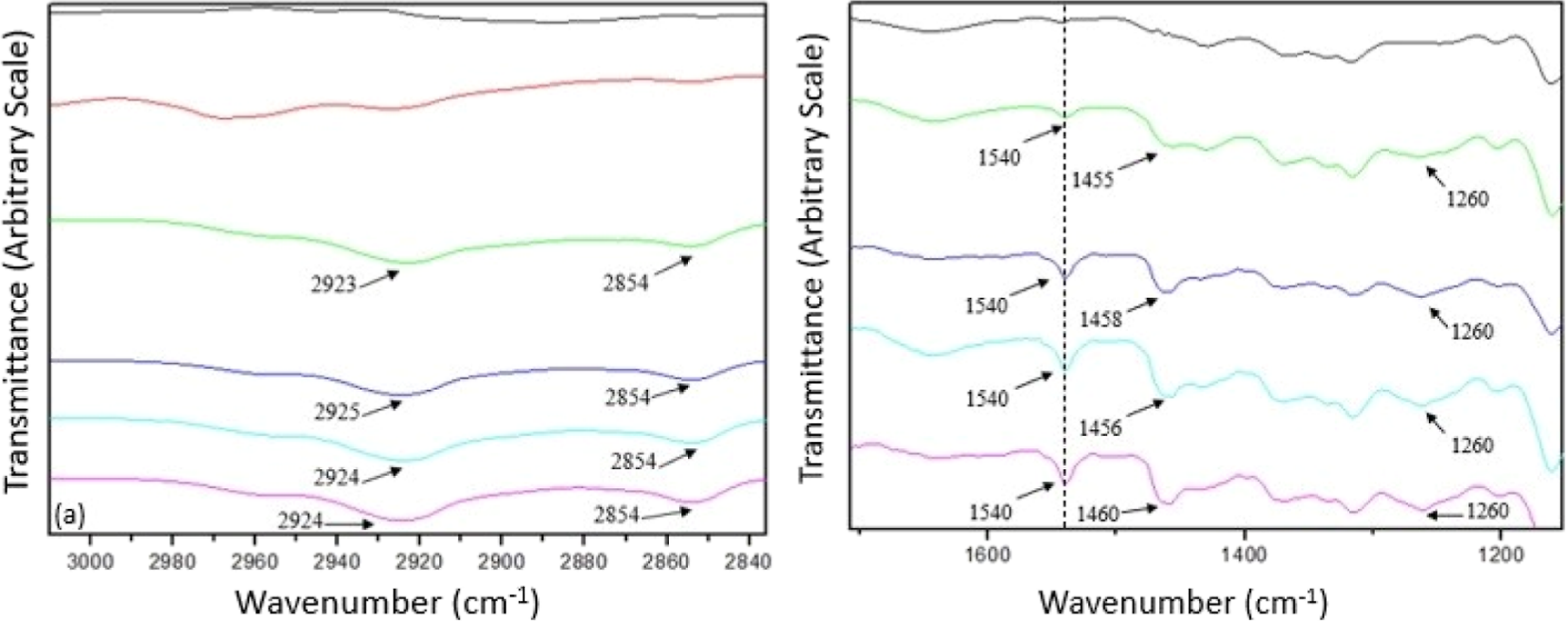
FTIR spectra with an expanded scale in regions (a) and (b): (black) CNFs; (red) 2AHS; (green) 1N; (blue) 3N; (sky blue) 5N; (violet) 7N.

The spectra regions (a) and (b) from Figure 2 are expanded in Figure 3a,b, respectively.

The success of the reaction is evidenced by the increased intensity of the methylene group grafted into the CNFs, and bands near 3000 cm^–1^ and 2850 cm^–1^ represent CH_2_ asymmetric and symmetric stretching,^29,30,32,33^ respectively, as shown in Figure 3a.

Additionally, a new low-intensity band at 1540 cm^–1^ (Figure 3b) corresponds to the angular deformation of the N–H group, further supporting the successful reaction. A noticeable increase in the band near 1460 cm^–1^, associated with in-plane angular deformation of the CH_2_ group, is also observed. A soft band at 1260 cm^–1^ attributed to C–N stretching is present in the modified CNFs but absent in the unmodified sample.^34^

### Elemental Analysis

The results from EA and the DS calculated by using eq 1 are presented in Table 2.

**Table 2:** EA of the Samples

samples	C (%)	H (%)	N (%)	DS
CNFs	41.69	8.69	0.00	0.00
1N	41.53	5.89	0.10	0.012
3N	45.35	6.90	0.45	0.053
5N	39.77	5.64	0.06	0.007
7N	42.43	5.93	0.11	0.013

The DS and N % values for reactions introducing amino groups into nanocellulose chains were relatively low, consistent with previous findings: Jakubovic (1960)^22^ (N % = 0.09–1.59), Pahimanolis et al. (2011)^35^ (N % = 0.24–0.26), Filpponen et al. (2011)^36^ (N % = 0.79), and Akhlaghi et al. (2015) (N % = 0.9).^25^ Saini et al. (2017)^26^ introduced amino groups onto nanofibrils using a three-step reaction process and found a similar nitrogen percentage of 0.4 with 3-aminopropyl trimethoxysilane. The reaction described in this study was performed in a single step.

The DS and N % values obtained in the present study are intermediate compared with those reported in the literature. This discrepancy may result from unequal functional group distribution, side reactions (e.g., competition between excess alkali and alkaline cellulose for the etherifying agent), or steric hindrance between the reagents during the reaction.^20,22,36−38^ Katsura (1992)^39^ highlighted the challenges of accurately determining DS values, particularly in ionic polysaccharides, which may also contribute to the observed variation.

### Atomic Force Microscopy

**Figure 4:**
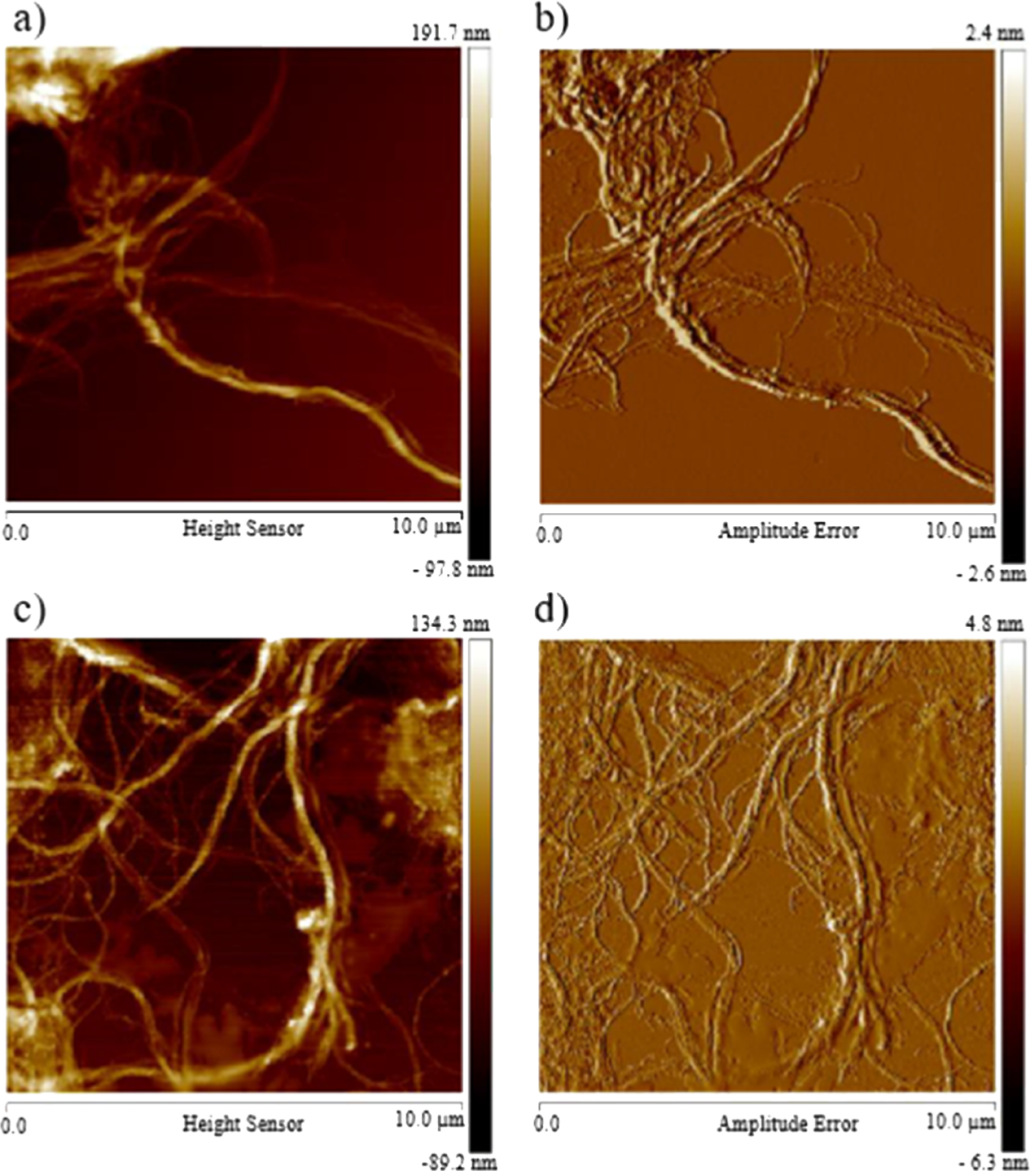
AFM analysis of pristine and modified 3N samples in relief (a,c) and amplitude (b,d).

AFM images, shown in Figure 4, reveal that the samples are organized as bundles of CNFs. Figure 4a,c displays the relief images, while Figure 4b,d depicts the amplitude images of CNFs before and after chemical modification (3N).

The modified CNFs exhibit greater dispersion compared with the unmodified samples, as observed in the images. The measured diameter ranges for pristine, 1N, 3N, 5N, and 7N samples were 0.34–63.53 nm, 0.03–29.88 nm, 0.19–44.15 nm, 0.28–49.75 nm, and 0.12–19.12 nm, respectively (Table 3). This enhanced dispersion and reduction in diameter can be attributed to the decreased intensity of hydrogen bonds, resulting from the grafting of the ethylamine group onto the polymeric chain.

**Table 3:** Diameter Distribution of the CNFs

diameter (nm)	frequency (%)				
	pristine	1N	3N	5N	7N
<1	8	14	8	6	20
1–10	50	66	60	62	70
10–20	26	14	12	24	10
20–30	6	6	4	4	0
30–40	4	0	12	2	0
>40	6	0	4	2	0

**Figure 5:**
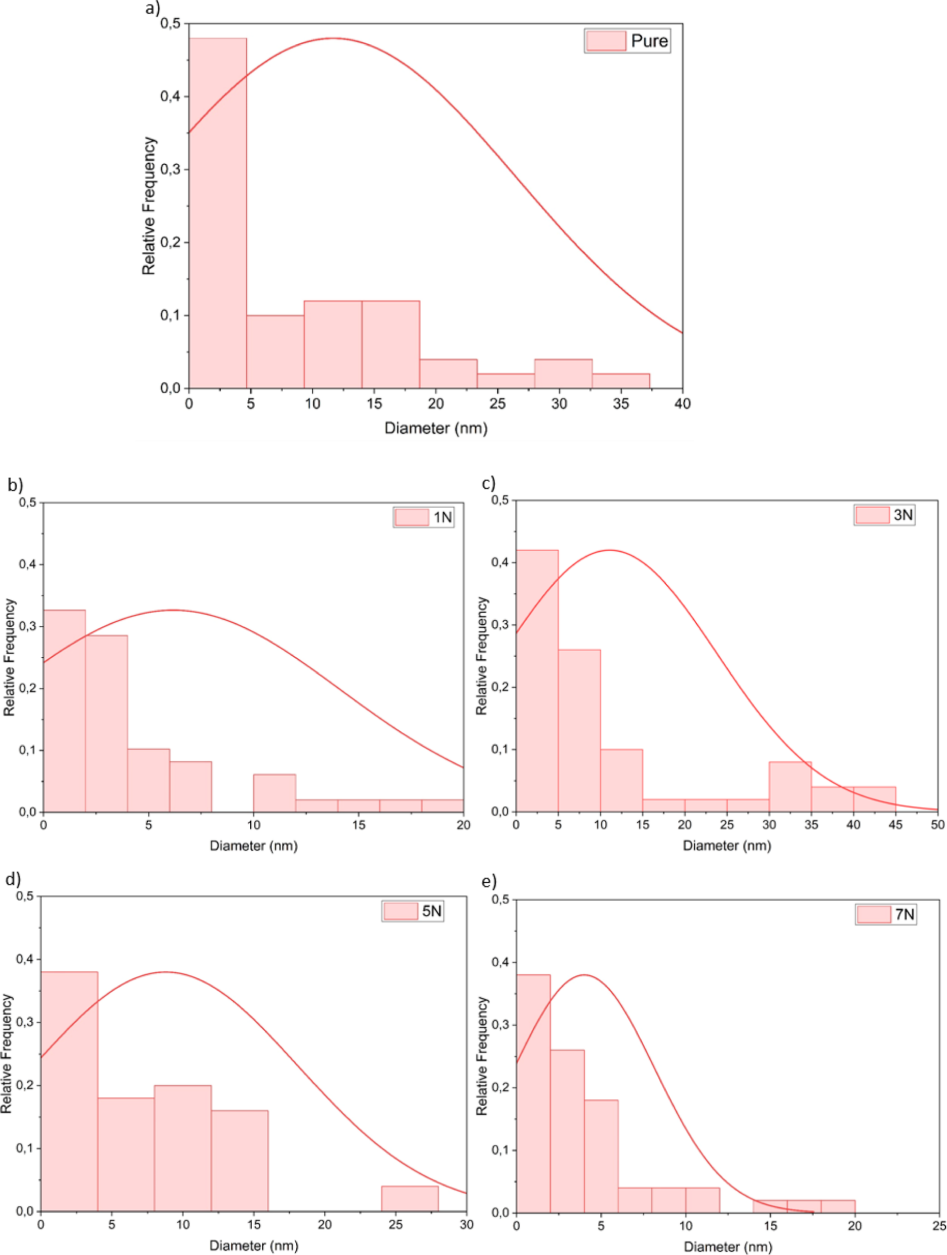
Histograms diameter versus relative frequency: (a) pure, (b) 1N, (c) 3N, (d) 5N, and (e) 7N.

The diameter distributions of the unmodified and modified CNFs are listed in Figure 5.

It is important to note that the morphology of CNFs can vary significantly depending on the source material and the method used to obtain them.^4,16,40^ Reported diameter ranges include 2–100 nm (Dufresne 2019);^4^ 3–5 nm (Pinto et al. 2019);^41^ 5–50 nm (Nechyporchuk et al. 2016);^16^ and 3–4 nm (Isogai et al. 2011).^42^ In the Supporting Information are presented the micrographs of others samples.

### X-ray Diffraction

**Figure 6:**
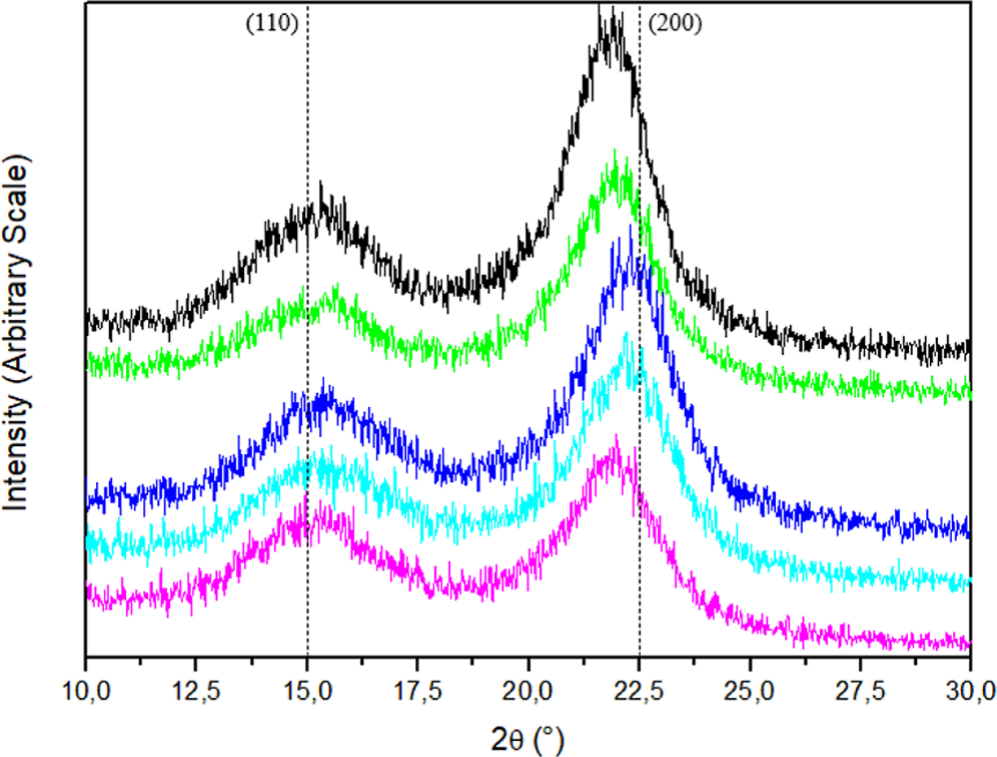
X-ray diffractograms: (black) CNFs; (green) 1N; (blue) 3N; (sky blue) 5N; (violet) 7N.

The diffractograms exhibit a valley at approximately 18°, associated with the amorphous phase, and both peaks at 15° and 22.5°, corresponding to the (110) and (200) crystallographic planes, respectively. These features confirm that cellulose has a type I structure. To evaluate the CI of the CNFs samples before and after chemical modification, Segal’s method (eq 2) was employed, which relates the peak maximum intensity peak to the adjacent valley, as shown in Figure 6.

The semicrystalline structure of the CNFs was preserved after modification; however, a decrease in CI was observed with increasing reaction time and temperature. This reduction in crystallinity is attributed to the incorporation of ethylamine groups inserted into nanocellulose, which disrupts the polymer chain’s ordered arrangement due to the bulkier size of the ethylamine groups compared to hydroxyl groups. Additionally, the decrease in CI may be attributed to swelling processes that occur in various solvents under acidic and basic conditions.^27,42,43^ The results of the CI are presented in Table 4.

**Table 4:** CI Values for the Samples

samples	2θ (deg)		CI (%)
	minimum	maximum	
CNFs	18.16	21.58	80.77
1N	18.53	21.94	80.27
3N	18.86	22.3	77.47
5N	18.38	22.2	74.83
7N	18.96	21.98	71.77

### Thermogravimetry

**Figure 7:**
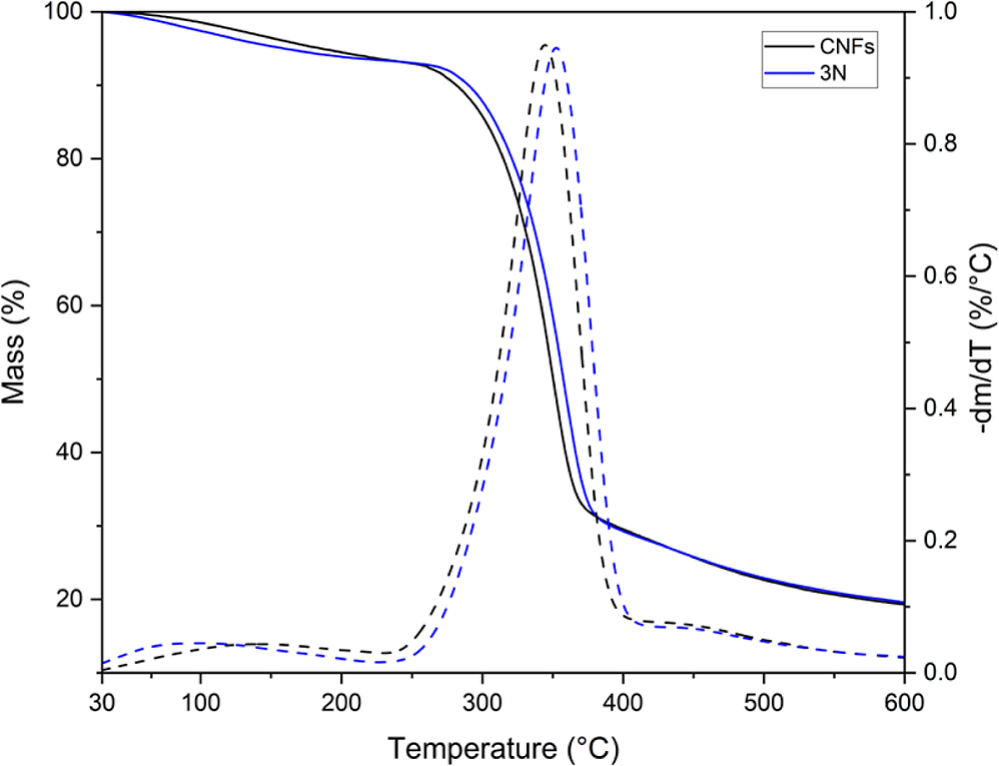
TG and dTG curves of pristine CNFs and the modified 3N sample.

Figure 7 presents the TG and dTG curves for the unmodified CNFs and 3N sample (the sample exhibited the highest N % yield). The TG and dTG curves for the other samples are provided in the Supporting Information.

From the thermal events observed in Figure 7, the data summarized in Table 5 was complied, detailing water evaporation and cellulose degradation. Both samples exhibited two distinct thermal events. The first, occurring between 27 and 150 °C, corresponds to water evaporation, resulting in a weight loss (WL) of approximately 8%. The second event, observed between 230 and 400 °C, is associated with cellulose decomposition, with a WL of approximately 66% for both samples.^28,45,46^^28,44,45^ The initial degradation temperature (*T*_onset_) showed a slight decrease, consistent with findings reported in the literature.^46−49^

**Table 5:** Thermal Events Observed in All Samples

sample	water evaporation			cellulose thermal degradation			
	*T*_onset_ (°C)	*T*_max_ (°C)	WL	*T*_onset_ (°C)	*T*_max_ (°C)	*T*_end_ (°C)	WL
CNF	30.4	138.8	6.5	229.4	344.1	425.8	65.9
1N	25.1	77.6	5.1	228.7	361.1	440.0	67.1
3N	27.2	96.3	6.6	227.5	352.6	430.9	66.3
5N	30.6	89.8	5.2	233.1	354.8	418.5	62.6
7N	25.7	97.4	6.5	235.8	354.4	432.7	65.0

### Differential Scanning Calorimetry

**Figure 8:**
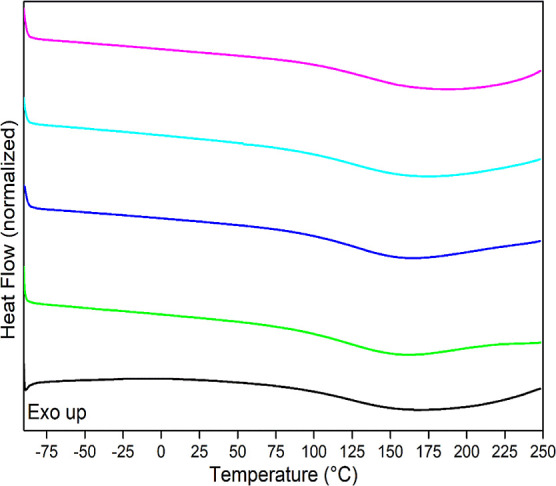
DSC curves of all samples: (black) CNFs; (green) 1N; (blue) 3N; (sky blue) 5N; (violet) 7N.

Figure 8 presents the DSC curves for unmodified and modified CNFs.

All samples exhibited endothermic events, with an onset temperature around 90–100 °C and an average temperature of approximately 160–170 °C, corresponding to sample dehydration. This behavior aligns with observations reported by de Menezes et al. 2009,^46^ where modified cellulose samples lacked well-defined melting peaks. Similarly, nanocellulose samples analyzed by Morán et al. 2008^44^ showed no melting peaks up to 250 °C, with dehydration occurring in the range of 30–140 °C. Comparable findings were also reported by Hou et al. 2009^50^ and Tozluoglu et al. 2017.^51^

Table 6 summarizes the initial temperature (*T*_onset_) and the average temperature (*T*_P_) for the dehydration event observed.

**Table 6:** *T*_onset_ and *T*_p_ of All Samples

samples	pure	1N	3N	5N	7N
*T*_onset_ (°C)	95.24	91.37	94.32	96.72	101.40
*T*_p_ (°C)	154.85	156.70	156.10	157.99	168.10

Residual humidity was detected during the analysis, even after the sample was quenched at 120 °C for minutes, as seen in Figure 8. The chemical modification appeared to have no significant impact on the DSC results, with all samples exhibiting similar thermal behavior.

## Conclusions

In summary, CNFs were successfully chemically modified via the S_N_2 reaction using 2AHS as the reagent. Among the experimental conditions carried out, the best result was achieved with sample 3N, which involved a reaction time of 2 h at 50 °C. The success of the chemical modification was confirmed through FTIR-ATR analysis and the nitrogen content obtained by EA. Morphological analysis via AFM demonstrated that the CNFs morphology was preserved. XRD data revealed a decrease in the CI due to the insertion of aminoethyl groups into the polymer chain, which introduced structural disorder. For sample 3N, the inclusion of these groups resulted in a slight decrease in the *T*_onset_ values, as observed in the TG and DTG curves. DSC results indicated similar thermal behavior across all samples with thermal events primarily associated with dehydration and the initial decomposition of glycosidic bonds.

## Data Availability

The authors declare that all data generated or analyzed during this study are included in this published article and its Supporting Information files.

## Supporting Information Available

AFM (pristine, 1N, 3N, 5N, and 7N) and FTIR and TG (1N, 5N, and 7N) results (PDF)

## Notes

Human and animal rights: The authors declare that there are no animal studies or human participant involvement in the study.
