# Supplementary material for: Chemical Functionalization of Cellulose Nanofibrils with 2-Aminoethyl Hydrogen Sulfate
Source: ACS Omega. 2024 Dec 23;10(1):1122–30. doi: 10.1021/acsomega.4c08573 (PMC11740635; doi:10.1021/acsomega.4c08573)
Supplement: Supplementary file 1 — ao4c08573_si_001.pdf [file ao4c08573_si_001.pdf]

Supporting Information

Chemical functionalization of cellulose nanofibrils with 2-aminoethyl hydrogen sulfate

Marcus Felippe de Jesus Barros1, Samir Leite Mathias1, Henrique Solowej Medeiros Lopes1,3, Marcelo de Assumpção Pereira da Silva4,5, Robson Valentim Pereira2, Aparecido Junior de Menezes1*.

1 - Graduate Program in Materials Science - Federal University of São Carlos - UFSCar, 18052-780, Sorocaba - SP, Brazil.

2 - Multidisciplinary Institute of Chemistry - Federal University of Rio de Janeiro - UFRJ, 27930-560, Macaé - RJ, Brazil.

3 - Technological College of Sorocaba – Fatec, 18013-280, Sorocaba - SP, Brazil

4 - Institute of Physics of São Carlos - University of São Paulo - USP, 13566-590, São Carlos - SP, Brazil.

5 - Central Paulista University Center - UNICEP, 13563-470, São Carlos - SP, Brazil.

*** jrmenezes@ufscar.br**

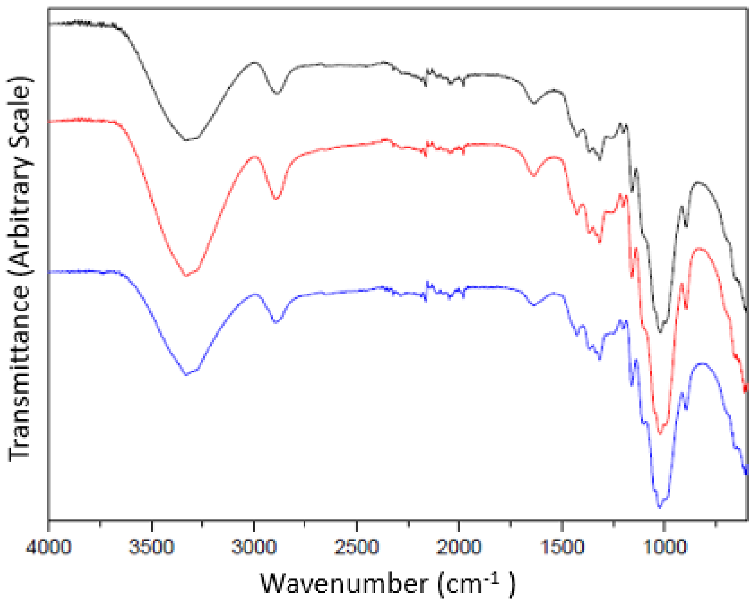

Fig. S1 FTIR spectra of chemical modification reactions under oxygen atmosphere and conditions: — 1h, 100 °C; — 2h, 100 °C; — 1h, 50 °C.

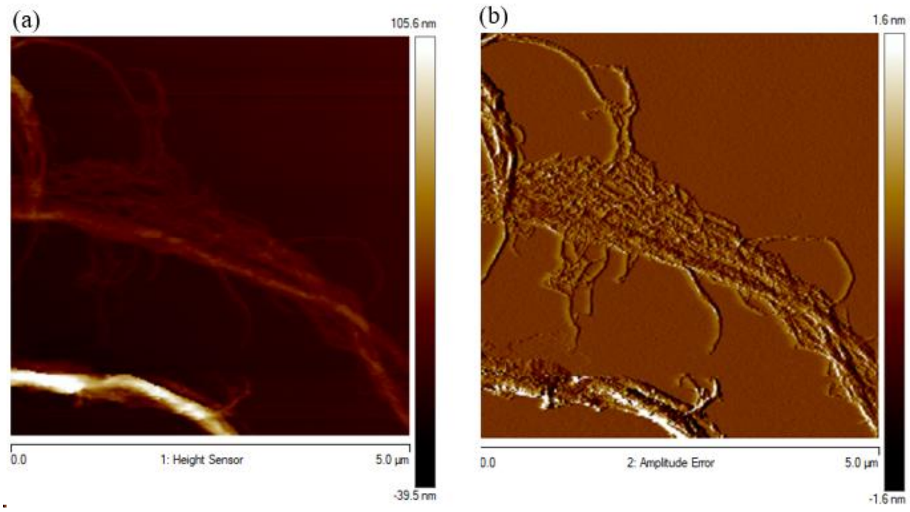

Fig. S2 AFM micrograph of the original sample: (a) height (b) amplitude.

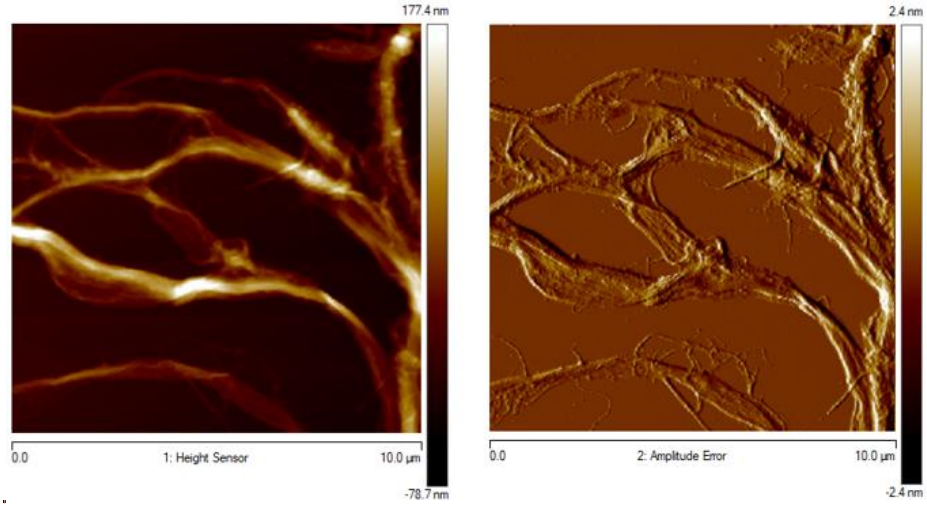

Fig. S3 AFM micrograph of the 1N sample: (a) height (b) amplitude.

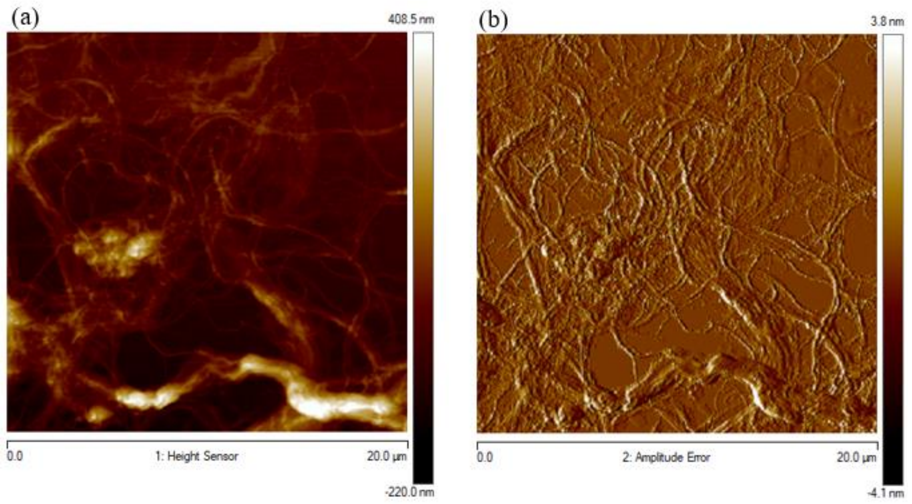

Fig. S4 AFM micrograph of the 3N sample: (a) height (b) amplitude.

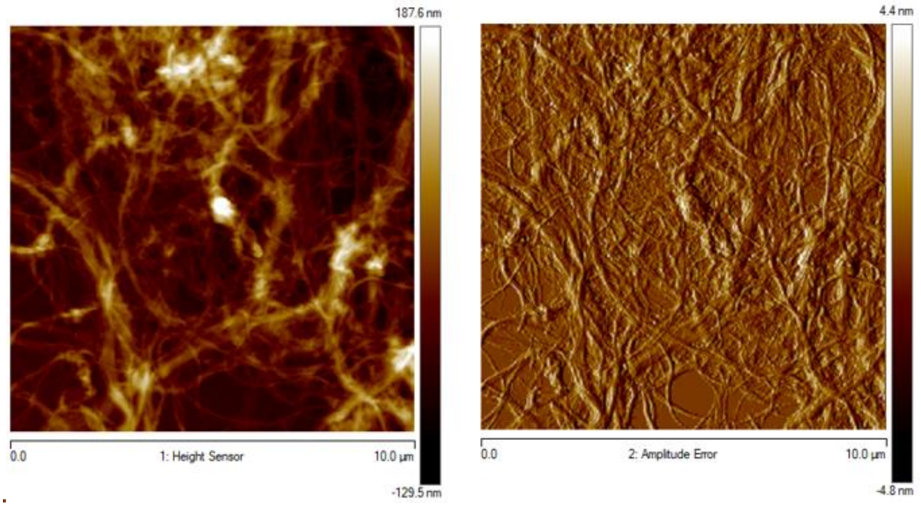

Fig. S5 AFM micrograph of the 5N sample: (a) height (b) amplitude.

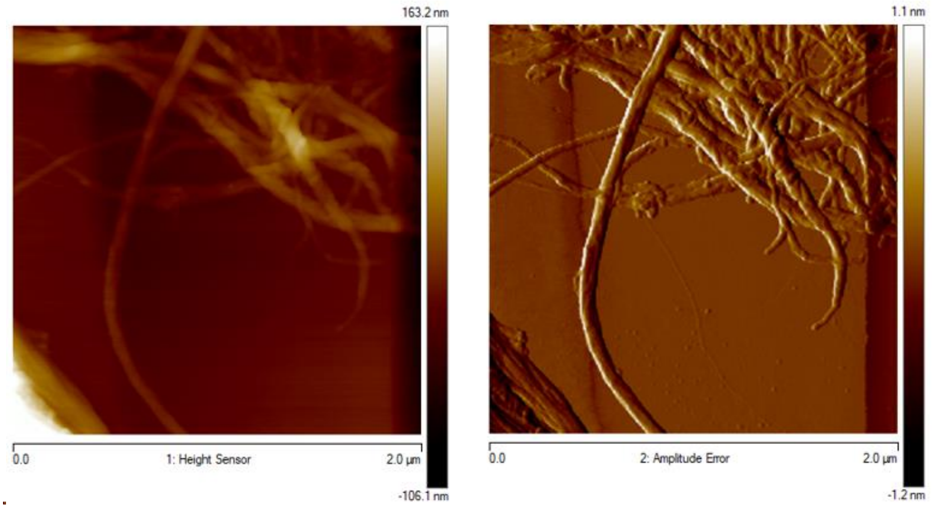

Fig. S6 AFM micrograph of the 7N sample: (a) height (b) amplitude.

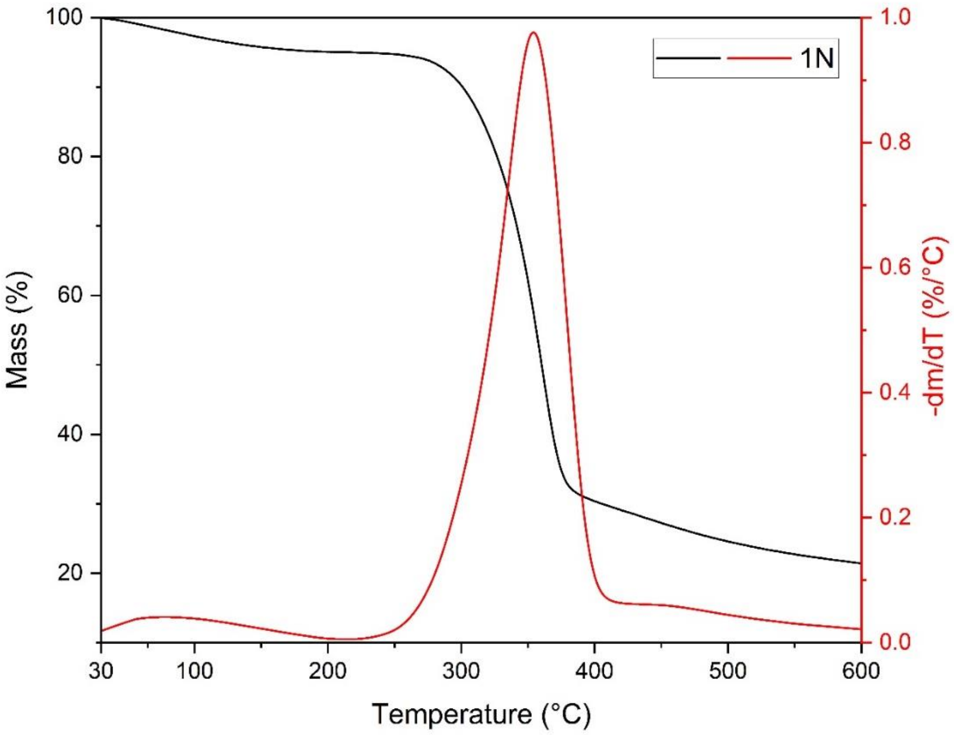

Fig. S7 TG and DTG curves for 1N sample.

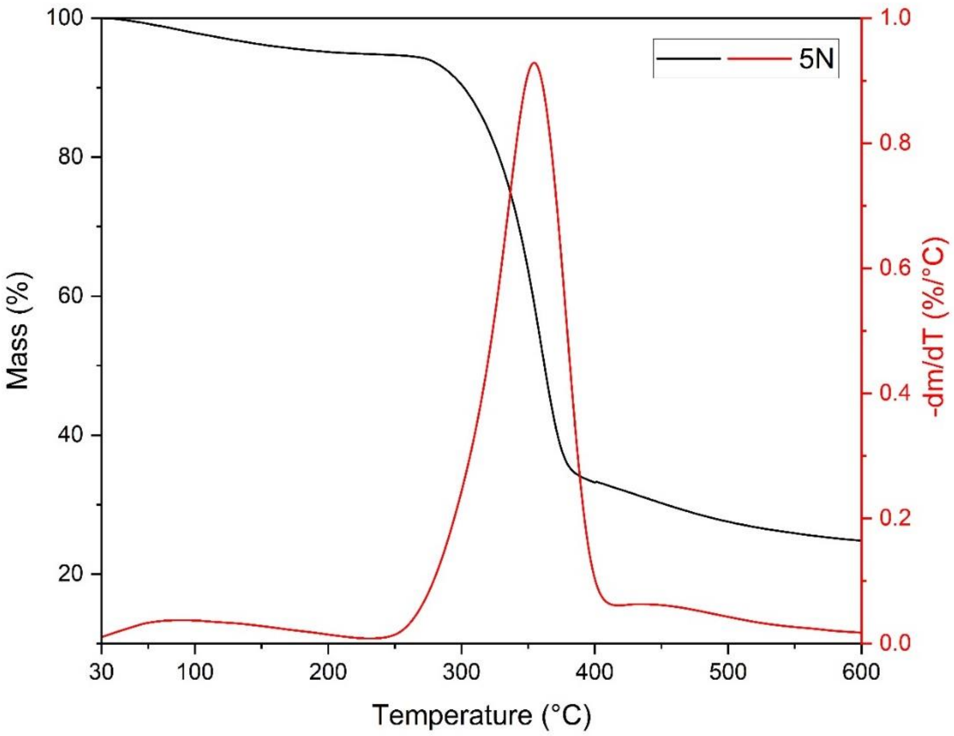

Fig. S8 TG and DTG curves for 5N sample.

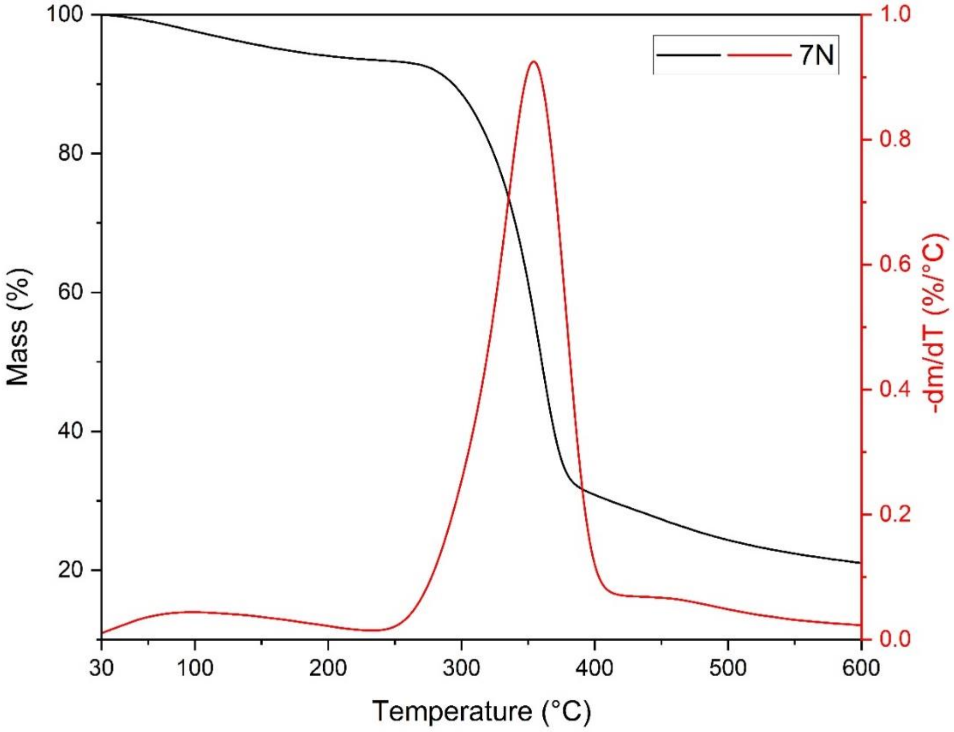

Fig. S9 TG and DTG curves for 7N sample.
